# Supplementary material for: Causal Relationship between Aging and Anorexia Nervosa: A White-Matter-Microstructure-Mediated Mendelian Randomization Analysis
Source: Biomedicines. 2024 Aug 16;12(8):1874. doi: 10.3390/biomedicines12081874 (PMC11351342; doi:10.3390/biomedicines12081874)
Supplement: Supplementary file 1 [file biomedicines-12-01874-s001.zip › Supplementary Figure/Figure S4/Singe SNP plot of MR analysis for TL to ubm-b-1818.pdf]

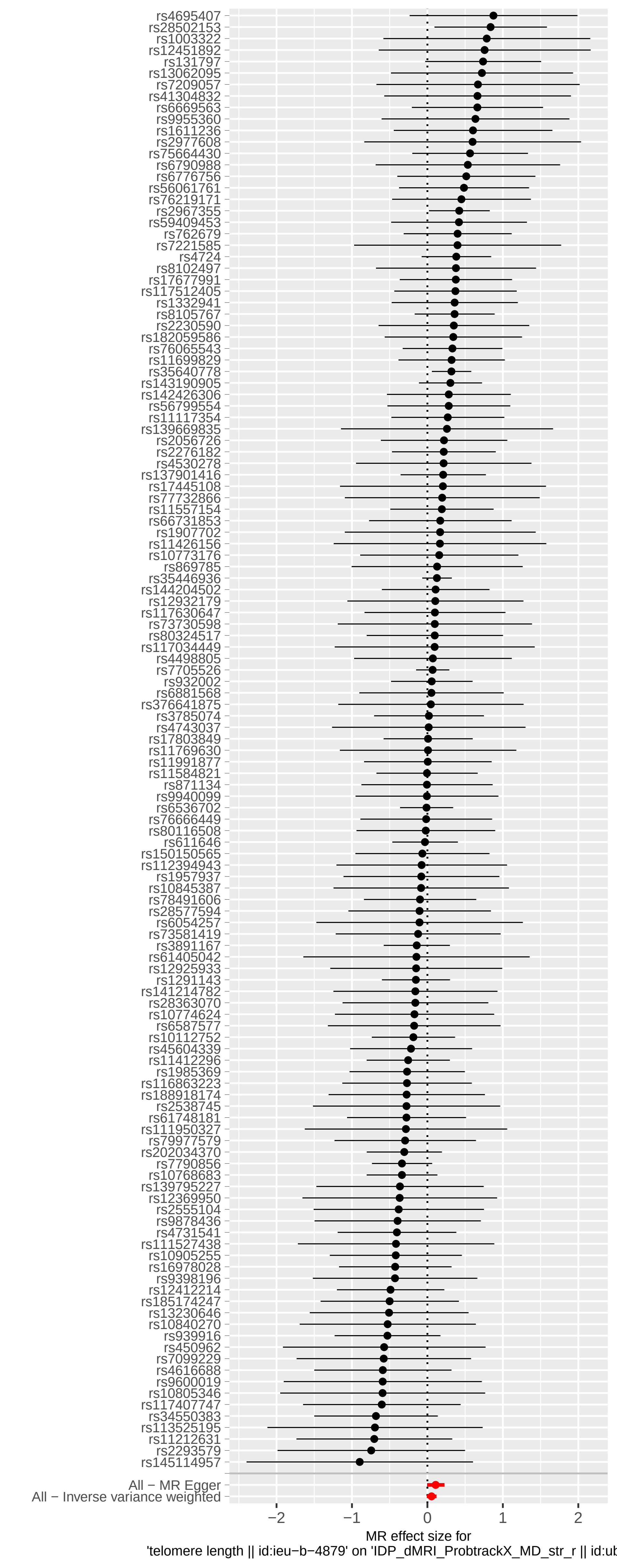
rs4695407
rs28502153
rs1003322
rs12451892
rs131797
rs13062095
rs7209057
rs41304832
rs6669563
rs9955360
rs1611236
rs2977608
rs75664430
rs6790988
rs6776756
rs56061761
rs76219171
rs2967355
rs59409453
rs762679
rs7221585
rs4724
rs8102497
rs17677991
rs117512405
rs1332941
rs8105767
rs2230590
rs182059586
rs76065543
rs11699829
rs35640778
rs143190905
rs142426306
rs56799554
rs11117354
rs139669835
rs2056726
rs2276182
rs4530278
rs137901416
rs17445108
rs77732866
rs11557154
rs66731853
rs1907702
rs11426156
rs10773176
rs869785
rs35446936
rs144204502
rs12932179
rs117630647
rs73730598
rs80324517
rs117034449
rs4498805
rs7705526
rs932002
rs6881568
rs376641875
rs3785074
rs4743037
rs17803849
rs11769630
rs11991877
rs11584821
rs871134
rs9940099
rs6536702
rs76666449
rs80116508
rs611646
rs150150565
rs112394943
rs1957937
rs10845387
rs78491606
rs28577594
rs6054257
rs73581419
rs3891167
rs61405042
rs12925933
rs1291143
rs141214782
rs28363070
rs10774624
rs6587577
rs10112752
rs45604339
rs11412296
rs1985369
rs116863223
rs188918174
rs2538745
rs61748181
rs111950327
rs79977579
rs202034370
rs7790856
rs10768683
rs139795227
rs12369950
rs2555104
rs9878436
rs4731541
rs111527438
rs10905255
rs16978028
rs9398196
rs12412214
rs185174247
rs13230646
rs10840270
rs939916
rs450962
rs7099229
rs4616688
rs9600019
rs10805346
rs117407747
rs34550383
rs113525195
rs11212631
rs2293579
rs145114957
All − MR Egger
All − Inverse variance weighted
−2
−1
0
1
2
MR effect size for
'telomere length || id:ieu−b−4879' on 'IDP_dMRI_ProbtrackX_MD_str_r || id:ub
